# Association between maternal smoking and duration of breastfeeding in very low birth weight preterm infants after discharge from a Neonatal Intensive Care Unit

**DOI:** 10.18332/tpc/194190

**Published:** 2024-10-29

**Authors:** Marta Costa-Romero, Andrea Mella-Bermudez, Tania Iglesias-Cabo

**Affiliations:** 1 Neonatal Intensive Care Unit, Cabueñes University Hospital, Gijon, Spain; 2 University of Oviedo, Oviedo, Spain; 3 Breastfeeding Association AELAMA, Madrid, Spain; 4 Statistical Consulting Unit, Scientific and Technical Services, Gijon, Spain

**Keywords:** tobacco, breastfeeding, premature infant health, very low birth weight preterm infants

**Dear Editor,**

Breastfeeding (BF) is regarded as the preferred method of feeding very low birth weight preterm (VLBW) infants in a Neonatal Intensive Care Unit (NICU) and following discharge. It has a well-documented role in protecting VLBW infants from serious complications during admission in an NICU, as well as reducing the likelihood of respiratory infections and potentially enhancing neurodevelopment^1-5^.

Tobacco use has been shown to alter the composition of breast milk (reducing macronutrients and altering immune status) and to decrease milk production. Both circumstances have clear implications for the infant’s health^6^. The aim of this study is to analyze the effect of smoking on the duration of BF after the discharge of VLBW infants.

This study was conducted based on the 1-year follow-up of VLBW infants born in a level III-A hospital in Spain between 2018 and 2022. The study received approval from the local ethics committee (CEImPA: 2023.459). We analyzed the rate of exclusive BF during NICU admission and after discharge up to 12 months of age, as well as clinical factors associated with the duration of BF. Mann-Whitney U test and linear models were employed. Univariate models were initially run, followed by a multivariate model that was simplified using a stepwise selection method. The data were analyzed using R V.4.2.1.

A total of 67 infants commenced BF on the first day of life. Follow-up was completed in 59 infants (6 infants died during admission and 2 relocated to other cities). Only 22 out of 59 (37%) infants continued BF at NICU discharge. By 12 months of age, only 10 out of 59 infants (16.9%) were still breastfeeding. Approximately 66% of the women who ceased BF were smokers, compared to 18% of those who continued (p=0.036). The linear model indicated that smoking was the main factor significantly associated with the duration of BF (Table 1). Specifically, if the mother smoked, the duration of BF after discharge decreased by 2.24 months.

**Table 1:** Linear models constructed to determine which factors are associated with BF duration after discharge from the NICU for VLWB infants born in level III-A hospital in Spain, 2018–2022 (N=59)

*Variables*	*P50 (P25–P75) Range*	*Mothers n*	*BF duration (months) Mean (SD)*	*Univariate Coef (95% CI), p*	*Multivariate Coef (95% CI), p*
**Gestational age** (weeks)	30 (28–31) 24.0–33.0		3.7 (4.1)	0.24 (-0.30–0.77), 0.375	
**Weight** (g)	1200 (875–1500) 610–1990		3.7 (4.1)	0.00 (-0.00–0.00), 0.473	
**Tobacco consumption**				-2.24 (-4.48 – -0.02), 0.048	-2.24 (-4.48 – -0.02), 0.048
No Yes		38 21	4.5 (4.7) 2.3 (2.6)		
**Previous BF**				0.97 (-1.54–3.48), 0.441	
No Yes		44 15	3.3 (4.0) 4.3 (4.2)		
**Previous children**				0.61 (-1.71–2.92), 0.602	
No Yes		38 21	3.4 (4.2) 4 (4.2)		
**Maternal age** (years)	36 (32–39) 19–48		3.7 (4.1)	0.05 (-0.14–0.23), 0.619	

Coef: coefficient. BF: breastfeeding. P50 (P25–P75): percentile 50 (percentile 27 – percentile 75). Univariate models were initially run, and then a multivariate model was simplified using a stepwise selection method. Given that tobacco consumption achieves statistical significance in the univariate model, it was included in the multivariate model, where it also proved to be significant.

Currently, early weaning from BF in preterm infants is a significant public health concern^5,7^. It has been noted that the rate of BF in preterm infants is considerably lower than in term infants, and that maintaining exclusive BF for more than a few months poses challenges for mothers of VLBW infants^5,7-9^. In fact, prematurity and maternal age appear to be risk factors for the early cessation of BF^1,5,10,11^.

The impact of smoking on the duration of lactation during NICU admission of VLBW infants^7^, after discharge of late preterm infants^4^, and on the initiation and maintenance of BF in term infants^9,11^, has been documented. However, to our knowledge, the relationship between smoking and the duration of BF after discharge of VLBW infants has not yet been published. During NICU admission, support from nurses and pediatricians may mitigate the influence of smoking^5^. Nevertheless, our data indicate that the effect of smoking persists post-discharge, underscoring the need for policies to support smoking mothers during and after NICU admission to enhance BF rates in preterm infants following discharge^4,5^. In conclusion, reducing smoking could increase BF rates in preterm infants after NICU discharge.

## Data Availability

Data sharing is not applicable to this article as no new data were created.
